# Expression of Potential Regulatory Genes in Abdominal Adipose Tissue of Broiler Chickens during Early Development

**DOI:** 10.1155/2014/318304

**Published:** 2014-01-16

**Authors:** Ann Bohannon-Stewart, Gary Kelley, Boniface Kimathi, Sri Harsha K. V. Subramanya, Joseph Donkor, Carl Darris, James Tyus, Ashley Payne, Shannon Byers, Dafeng Hui, Samuel Nahashon, Fur-Chi Chen, Michael Ivy, Xiaofei Wang

**Affiliations:** ^1^College of Agriculture, Human and Natural Science, Tennessee State University, Nashville, TN 37209, USA; ^2^Department of Biological Sciences, Tennessee State University, 3500 John A. Merritt Bou Levard, Nashville, TN 37209, USA

## Abstract

The identities of genes that underlie population variation in adipose tissue development in farm animals are poorly understood. Previous studies in our laboratory have suggested that increased fat tissue involves the expression modulation of an array of genes in broiler chickens. Of special interest are eight genes, *FGFR3, EPHB2, IGFBP2, GREM1, TNC, COL3A1, ACBD7*, and *SCD*. To understand their expression regulation and response to dietary manipulation, we investigated their mRNA levels after dietary manipulation during early development. Chickens were fed either a recommended standard or a high caloric diet from hatch to eight weeks of age (WOA). The high caloric diet markedly affected bodyweight of the broiler birds. mRNA levels of the eight genes in the abdominal adipose tissue were assayed at 2, 4, 6, and 8 WOA using RT-qPCR. Results indicate that (1) *FGFR3* mRNA level was affected significantly by diet, age, and diet:age interaction; (2) *COL3A* mRNA level was repressed by high caloric diet; (3) mRNA levels of *EPHB2, ACBD7*, and *SCD* were affected by age; (4) mRNA level of *TNC* was modulated by age:diet interaction; (5) changes in *GREM1* and *IGFBP2* mRNA levels were not statistically different.

## 1. Introduction

In chickens, quantitative trait loci (QTL) mapping studies have identified a number of loci in fat deposition [1–7]. In different mapping populations, the loci that contribute to fat deposition appear to be unique because different chicken strains inherited a unique set of alleles. Since QTL mapping studies are often conducted in F2 crosses or backcrosses, which have limited chromosomal crossovers, they often end up with broad chromosomal regions in which hundreds of candidate genes may potentially be responsible for the fat QTL. The identities of genes or regulatory elements for the inferred traits are thus unknown. On the other hand, candidate genes are often evaluated through association studies using DNA sequence variations such as single nucleotide polymorphism (SNP) and short tandem repeats [8–12]. Various alleles of quite a few of the evaluated genes were shown to increase fat deposition.

During early development, the chicken adipose tissue grows by both hyperplasia and hypertrophy [13, 14]. Hyperplasia depends on the proliferation of preadipocytes, since mature adipocytes do not multiply. Although there are few studies on preadipocyte proliferation in chickens [15–19], it is generally believed that its mechanism would be similar to that in mammals and other vertebrates, as almost all genes that play key roles in mammals are also found in the chicken. For example, lipogenic transcription factors such as PPARg and CCAAT/enhancer binding proteins are clearly expressed in the chicken preadipocytes and have similar roles in lipogenesis [15, 17, 19] as in other species. Many growth factors have also been shown to regulate adipocyte proliferation in chickens [13]. At least a portion of the genes involved in preadipocyte hyperplasia are also involved in adipocyte hypertrophy. PPARg not only induces preadipocyte proliferation and differentiation but also stimulate lipogenic gene expression in adipocytes [20].

There are obvious differences between mammalian and avian adipose tissues. The chicken does not have GLUT4, the major protein that transports glucose across the plasma membrane in response to insulin. Thus, other glucose transporters must be in place of GLUT4 [21]. The synthesis of fatty acids takes place predominantly in the liver, with adipocytes contributing a small portion of fatty acid synthesis to the stored lipids [22], which is in sharp contrast to that in many mammals. It is largely questionable as to whether there is a leptin gene in the chicken [23], although the chicken leptin receptor does exist and can be experimentally demonstrated [24].

Numerous gaps exist in our knowledge regarding chicken adipose tissue development, particularly the molecular events that lead to differential fat deposition given the same feeding. We have previously studied gene expression in adipose tissue of chickens fed the same ration with similar bodyweight but differential abdominal fat weights and fat percentages. This study revealed a number of differentially expressed genes in abdominal fat tissue between fat and lean chickens (reported separately). It is of special interest to evaluate the regulatory mechanisms of these differentially expressed genes so that their roles and regulatory mechanisms in fat deposition are better understood. Here, we have selected a group of the differential genes that likely play a regulatory role or are target of regulation in different metabolic or development status. The selected genes include four encoding signaling proteins (*FGFR3* encoding fibroblast growth factor receptor 3, *EPHB2* encoding ephrin type-B receptor 2, *GREM1* encoding gremlin 1, and *IGFBP2* encoding insulin-like growth factor binding protein 2), two extracellular matrix genes (*COL3A1* encoding collagen, type III, alpha 1, and *TNC *encoding cytotactin or tenascin-C), and two metabolic genes (*ACBD7* encoding acyl-CoA-binding domain containing 7 and *SCD* encoding stearoyl-CoA desaturase). We hypothesize that these genes are regulated either by nutritional factors or by developmental programming in chickens. To test this hypothesis, broiler chickens were treated with a high caloric diet (HCD) or recommended standard diet (RSD) for 8 weeks; then mRNA levels were analyzed in abdominal fat tissue at 2, 4, 6, and 8 weeks of age (WOA). Here, the results of this study are reported.

## 2. Materials and Methods

### 2.1. Animals and Dietary Treatment

New hatched broiler chickens (113 males and 113 females, commercial generation) were purchased from Ideal Poultry (Texas, USA) and reared at Tennessee State University in the Frank A. Young Poultry Research Plant. Birds of each sex were assigned at random to two dietary treatments: RSD or HCD. There were six replicates (*n* = 9) for each treatment of each sex. The diets were corn-soy-based. RSD contained 21% crude protein, 3040 kcal/kg of metabolizable energy (ME), formulated per National Research Council recommendations for broiler chickens. HCD contained 23% crude protein, 3340 ME, formulated based on preliminary experimental rations designed to induce obesity in growing broilers. The use of animals was approved by Tennessee State University Institutional Animal Care and Use Committee (IACUC).

All birds were weighed weekly. At 2, 4, 6, and 8 WOA, one chicken from each replicate was sacrificed. Samples of abdominal fat pad and fat around the gizzard were collected, weighed, placed in liquid nitrogen for direct freeze, and then transferred to −80°C until RNA isolation.

### 2.2. RNA Isolation and RT-qPCR

Abdominal adipose RNA of male chickens (*n* = 6 per age group) was extracted with RNeasy Lipid Tissue Midi kit (Qiagen). RNA concentrations were measured with a NanoDrop spectrophotometer (NanoDrop Technologies, Wilmington, DE). All samples were analyzed with Experion RNA StdSens analysis kit to determine the quality. PCR primers were designed using Primer Express 2.0 (Applied Biosystems; see Table 1 for primer information). All RT-qPCR assays were conducted using QuantiTect SYBR Green RT-PCR kit (Qiagen). Reaction was done in 20 *μ*L containing 50 ng of total RNA and 0.4 *μ*M of each primer. Thermal cycles contained one cycle of pre-incubation at 50°C for 10 minutes and 95°C for 15 minutes, 35 cycles of amplification (95°C for 15 seconds and 60°C for 60 seconds). Primers were validated by melting curve analysis, standard curve, and nontemplate control reactions. For standard curve analysis, an RNA pool was made, serial-diluted to 0.08, 0.39, 1.56, 6.25, 25, 50, and 100 ng/*μ*L, and measured again with spectrophotometer. Each concentration was analyzed in duplication with RT-qPCR to determine amplification efficiency.

### 2.3. Data Analysis

*t*-test was used to compare mean bodyweight between groups. ANOVA and Fisher post hoc test were used to analyze RT-qPCR data.

## 3. Results

### 3.1. Effect of Dietary Treatment on Animal Growth

**Figure 1:**
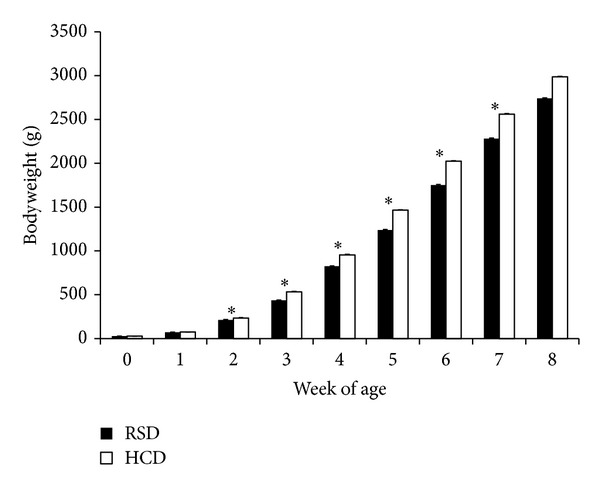
Live bodyweight (g) chickens (males and females) fed RSD or HCD. Bars represent bodyweight (mean ± SE), and asterisks denote significant differences (*P* < 0.01) in bodyweight at the same age between HCD and RSD.

Birds fed HCD had significantly higher bodyweight (*P* < 0.01) than those fed RSD at 2 through 7 WOA (Figure 1). At 8 WOA, birds fed HCD were still larger than those fed RSD, though not statistically significant, which may be due to the fact that there were fewer birds in each group at this age. At 1 WOA, there were no significant differences between treatments, probably due to not having enough time to accumulate bodyweight.

Bodyweight gain (BWG) for animals that were fed HCD and RSD is presented in Table 2, in which BWG was grouped by sex. Both males and females on HCD established greater gains than their counterparts on RSD. For the male group, birds on HCD gained significantly more weight than those on RSD during weeks 2–5. However, there were no significant differences between the two groups during 6, 7, and 8 WOA. This is interesting in that, in the final two weeks, males on RSD did display higher weight gains than males on HCD. During weeks 2 and 3, females on HCD displayed significantly higher BWG (*P* < 0.05) than females on RSD. A similar result was also observed during week 6. Much like in the males, during the last 2 weeks, there were greater BWG for birds on RSD. The data indicates that the HCD led to an increase in BWG in both sexes.

When different diet groups of the same sex were compared, bodyweights showed no significant difference in both male and female chicks at hatch (Table 3). At 1 and 2 WOA, female birds fed HCD had a higher bodyweight than females fed RSD (*P* < 0.05). This suggests that the HCD affected the females at an earlier age than the males because the males had no significant differences between diet groups at 1 and 2 WOA. At 3 and 4 WOA, birds fed HCD had a higher bodyweight than those fed RSD (*P* < 0.01) in both males and females. At 5 WOA, males on HCD were heavier that those on RSD with *P* < 0.01, while females on HCD were also heavier than their counter part on RSD with *P* < 0.05. At 6 WOA, both males and females fed HCD were significantly different from those fed RSD at *P* < 0.01. At 7 WOA, males had significant differences at *P* < 0.05 level and females had *P* < 0.01 significant differences between diet groups. At 8 WOA only females had a significant difference at *P* < 0.05.

When mean bodyweights at the same age on the same diet were compared, males appeared heavier than the females through most ages, though not statistically significant; however, at 3 WOA, the females were heavier than males in both diet groups. At 5 WOA, males on HCD showed a significant difference (*P* < 0.05) over females (Table 3).

### 3.2. Effect of Dietary Treatment on Abdominal Fat Content

**Figure 2:**
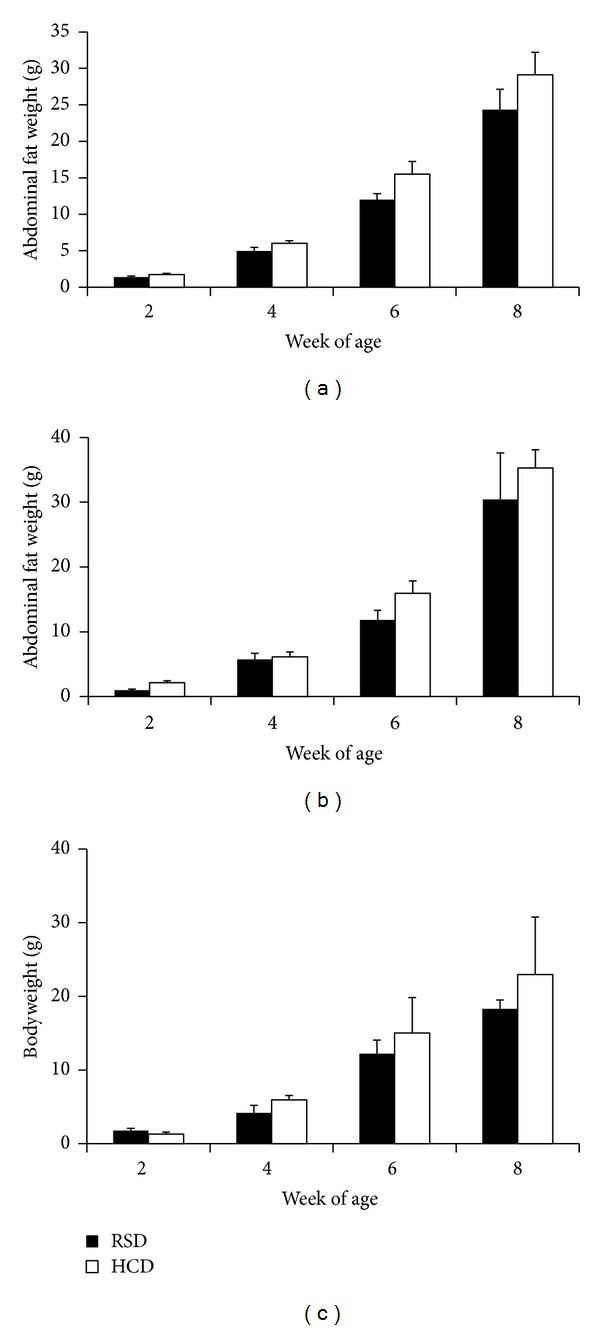
Abdominal fat weights (mean ± SE, g). (a) Comparison between HCD and RSD, with males and females combined (*n* = 12). (b) Female abdominal adipose tissue weight (*n* = 6). (c) Male abdominal adipose tissue weight (*n* = 6).

Figure 2 shows comparisons of abdominal fat weights between birds fed RSD and HCD. It appeared that birds fed HCD had accumulated more abdominal fat on average than those fed RSD, though the differences were either statistically marginal or insignificant, likely due to the large variations among individuals. This observation holds true, regardless if males and females were examined separately (Figures 2(b) and 2(c)) or combined (Figure 2(a)). Likely, statistically significant differences in fat deposition between HCD and RSD birds could be demonstrated when more birds are dissected.

**Figure 3:**
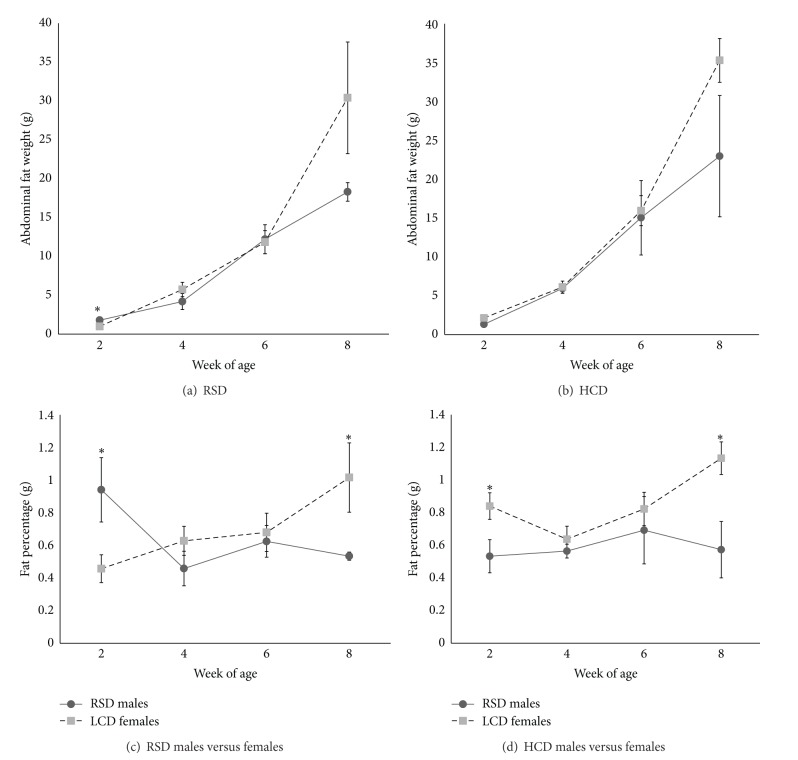
Comparison of abdominal adipose tissue weight (mean ± SE, g) and percentage between males and females. (a) Abdominal fat weight on RSD. (b) Abdominal fat weight on HCD. (c) Abdominal fat as the percentage of bodyweight in chickens on RSD. (d) Abdominal fat as the percentage of bodyweight in chickens on HCD. *n* = 6. Asterisk denotes significant difference (*t*-test, *P* < 0.05).

When compared between males and females on the same diet, males had significantly more fat tissue than females at 2 WOA on RSD (*P* < 0.05), as measured in absolute amount (g) and in bodyweight percentage (Figures 3(a) and 3(c)). By 8 WOA, female chickens appeared to have accumulated more abdominal adipose tissue than males when both were fed RSD (Figure 3(a)) and had 12.3 g more than males when both were fed HCD (Figure 3(b)), though the differences were not statistically significant. The differences became statistically significant when fat tissue was calculated as percentage of bodyweight (Figures 3(c) and 3(d)). There were no significant differences in HCD and RSD between sexes at 4 and 6 WOA (Figures 3(a), 3(b), 3(c), and 3(d)).

### 3.3. Effects of Dietary Treatment and Age on mRNA Levels

**Figure 4:**
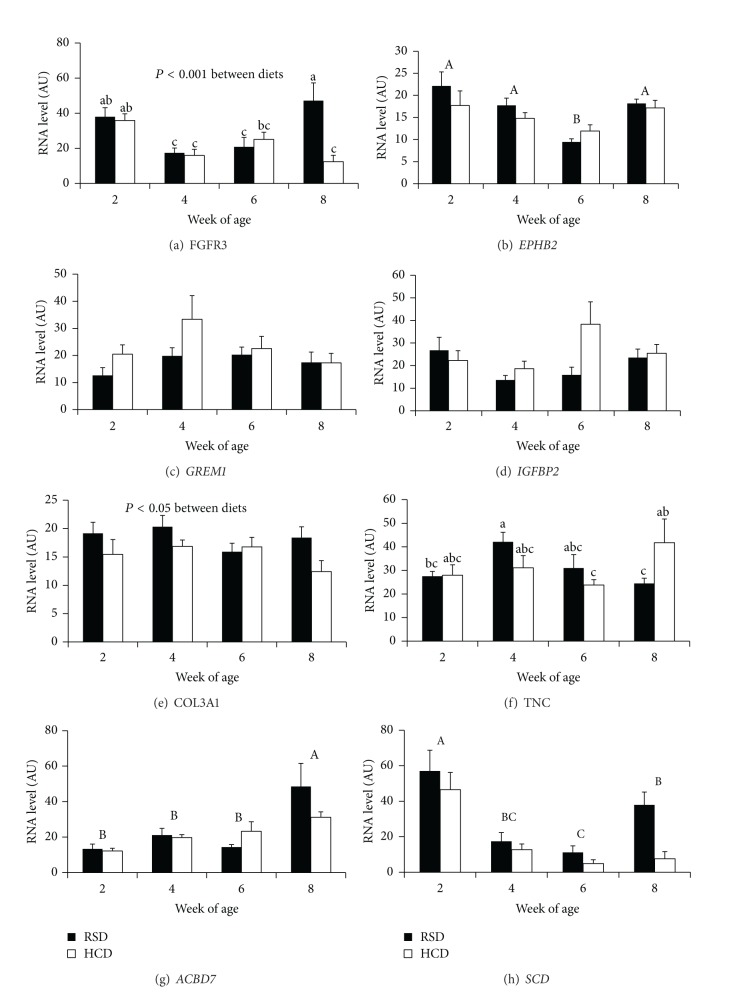
Levels of mRNA in adipose tissue of male broiler chickens during 2–8 WOA. ANOVA and Fisher LSD post hoc test were used to examine statistical significance (duplicated determination, *n* = 6 for *GREM1*, *IGFBP2*; *n* = 5 for all other genes). Lower case scripts mark significant difference in mRNA levels (mean ± SE) grouped by the same age and diet, where bars without a common lower case script were different from one another (*P* < 0.05). Capital scripts mark significant differences among mRNA levels grouped by age, with which mRNA levels without a common capital script were significantly different from one another.

Four signaling related genes were examined for mRNA level. *FGFR3* displayed significant differences among both age and dietary groups as well as age by diet interaction. At 2 WOA, *FGFR3 *mRNA was significantly higher than that at 4 and 6 WOA. At 8 WOA, *FGFR3* mRNA levels were significantly elevated in birds fed RSD (Figure 4(a)). *EPHB2* showed an age-dependent decrease in expression level (*P* < 0.001) from 2 to 6 WOA and then increased at 8 WOA, but diet did not affect *EPHB2* expression, nor did the interaction between age and diet (Figure 4(b)). For *GREM1* and *IGFBP2*, dietary treatments did not affect their mRNA level, nor did age (Figures 4(c) and 4(d)).

Two genes encoding extracellular matrix were examined. *COL3A1 *mRNA showed significant differences between groups fed HCD and RSD (*P* < 0.05) but not among age nor among age by diet interactions. In general, birds fed HCD tend to express less *COL3A1* than those fed RSD (Figure 4(e)). *TNC* expression displayed significant differences in age by diet interaction (*P* < 0.05) but not age nor diet alone (Figure 4(f)).

Two metabolic genes, *ACBD7 *and *SCD,* were also examined. *ACBD7* showed an age-dependent increase in expression level (*P* < 0.001), but diet did not affect *ACBD7* expression, nor did the interaction between age and diet (Figure 4(g)). *SCD *showed an age-dependent decrease in expression level (*P* < 0.001) until 8 WOA, but diet did not affect *SCD *expression, nor did the interaction between age and diet (Figure 4(h)).

## 4. Discussion

In this experiment, birds fed HCD gained significantly greater bodyweight than those fed RSD. Abdominal fat weight in chickens fed the HCD appeared higher than that in chickens fed RSD, though the differences were not significant. Nevertheless, dietary manipulation caused significant phenotypic changes in chickens during the period studied. In terms of gene expression, mRNA levels of three genes were markedly affected by either dietary manipulation (i.e., *COL3A1* and *FGFR3*) or the interaction of dietary manipulation and age (*TNC* and *FGFR3*). *COL3A1* expression was, in general, reduced in chickens on HCD. The mRNA level of *TNC* was significantly higher in chickens fed HCD than those fed RSD at 8 weeks of age. In general, dietary manipulation affected chicken growth and fat deposition, at least partially by modulation of *FGFR3, COL3A1,* and *TNC* expression.

Among the eight genes examined in this study, five genes, *FGFR3, EPHB2, TNC, ACBD7, *and* SCD, *exhibited developmental changes in expression level. *FGFR3* mRNA level was decreased from 2 to 4 WOA and then kept approximately steady until 8 WOA when there were significant age by diet interactions (Figure 4(a)). *TNC* mRNA level was affected as a result of age:diet interaction (Figure 4(f)). *EPHB2 *mRNA level was first lowered from 2 to 6 WOA and thereafter increased from 6 to 8 WOA. *TNC *expression displayed an age-dependent response to dietary manipulation. At 8 WOA, chickens fed RSD had significantly lower *TNC *mRNA level than those fed HCD. The mRNA level of *ACBD7* increased steadily and that of *SCD* decreased steadily during the first 8 weeks of development. These data indicate that developmental programming has the most significant effect on the expression of these genes in chicken adipose tissue. At least during early age, the growth of chicken adipose tissue involves preadipocyte proliferation and size increase. There are many different cell types involved in the remodeling of adipose tissue. Thus, whether the expression changes of these genes occurred in a specific cell type or in all cell types remains to be clarified.

*FGFR3* is an important regulator of bone growth and has a strong proliferative effect on cancer (reviewed in [25]). The role of both *FGFR3* and *GREM1* in adipose tissue is unknown. Considering their mitogenic activities, it is plausible to assume that these genes are necessary for preadipocyte proliferation and vascular development in adipose tissues. Our assay showed that *FGFR3* expression level decreased in the first few weeks. Whether this is associated with reduced adipocyte proliferation remains unclear.

*EPHB2* encodes the ephrin B2 receptor, a transmembrane protein involved in signaling [26]. Previous studies on *EPHB2 *function were mainly focused on its role in tumor growth [27–29] and brain development [30, 31]. To our knowledge, few studies have been conducted regarding *EPHB2* in adipose tissue. Our study shows that *EPHB2* is expressed in adipose tissue, where likely sources of *EPHB2 *expression include adipocytes, macrophages, and angiogenic cells. Macrophages and angiogenic cells have been shown to express *EPHB2*. Adipocytes also express *EPHB2*, as evidenced by the presence of *EPHB2 *transcripts in 3T3-L1 adipocytes in the Gene Expression Omnibus database. We found that *EPHB2 *mRNA level was gradually reduced in chicken adipose tissue during 2–6 WOA and then increased at 8 WOA. The reason for the mRNA level change is not clear. Since adipose tissue growth requires tissue remodeling, *EPHB2 *may play a role in the remodeling process.

The protein product of *GREM1* is required for early limb outgrowth and patterning, particularly in bone growth [32, 33]. It also plays a role in angiogenesis by acting as an agonist of the major angiogenic VEGFR2 [34]. In this study, *GREM1 *did not show any statistical difference among ages and diet, indicating that it is not regulated by these two factors during this developmental stage in chicken adipose tissue.

*IGFBP2* inhibits IGF-mediated growth and developmental rates [35]. IGF-binding proteins prolong the half-life of the IGFs and have been shown to either inhibit or stimulate the growth promoting effects of the IGFs on cell culture. They alter the interaction of IGFs with their cell surface receptors [36]. Studies have shown that *IGFBP2* genetic variation is associated with fat deposition in chickens [37–39]. However, the level of *IGFBP2* mRNA was unresponsive to diet and was also not affected by age in adipose tissue. The effect of *IGFBP2* on adipose tissue development does not involve the direct regulation of *IFGBP2* mRNA level in this tissue.

Tenascins and collagens are extracellular matrix proteins. Tenascin-C is a substrate-adhesion molecule in the glycoprotein family [40]. It helps to regulate cell proliferation, adhesion, and migration [41] in developing embryos. Other reports claim that tenascin-C is predominantly expressed during embryonic development and wound healing [42]. As an essential component of extracellular matrix, collagens not only play a supporting role for adipocytes but also regulate the development of adipose tissue by participating in signaling in mammals. For example, null-collagen VI expression is associated with increased adipose tissue [43]. On the other hand, disruption of collagens V and VI synthesis may cause impaired triglyceride accumulation in adipocytes [44]. Our study showed that *COL3A1* expression was altered by dietary manipulation. Birds fed RSD expressed higher levels of *COL3A1 *mRNA, suggesting that collagen III participates in and regulates the remodeling of adipose tissue in chickens.

*ACBD7* is a small 10 KD protein with acyl-CoA-binding (ACB) domain. The ACB domain consists of four alpha-helices arranged in a bowl shape with a highly exposed acyl-CoA-binding site. The ligand is bound through specific interactions with residues on the protein. This is by several conserved positive charges that interact with the phosphate group on the adenosine-3′ phosphate moiety, and the acyl chain is the middle of the hydrophobic surfaces of CoA and the protein [45]. Acyl-CoA-binding protein (ACBP) binds thiol esters of long fatty acids and coenzyme A in a one-to-one binding mode with high specificity and affinity. Acyl-CoAs are reported to play a large role as intermediates in fatty lipid synthesis and fatty acid degradation. Therefore, they play a part in the regulation of intermediary metabolism and gene regulation. The role of ACBP is believed to be an intracellular acyl-CoA transporter and pool former [46]. In our study we found a significant difference in age only, which seems to be a steady increase from 2 to 8 WOA.

In vertebrates *SCD*s are key enzymes involved in de novo monounsaturated fatty acid synthesis [47]. *SCD*s are responsible for forming a double bond in stearoyl-CoA. This is how the monounsaturated fatty acid oleic acid is produced from the saturated fatty acid stearic acid. *SCD* catalyzes a rate-limiting step in the synthesis of unsaturated fatty acids. The principal product of *SCD* is oleic acid, which is formed by desaturation of stearic acid. The ratio of stearic acid to oleic acid has been implicated in the regulation of cell growth and differentiation through effects on cell membrane fluidity and signal transduction [48–50]. *SCD*s also generate essential components of phospholipids, triglycerides, cholesterol esters, and wax esters [47]. In chickens, there is much reported evidence that *SCD* plays a potential role in the control of bodyweight and energy homeostasis, and the expression level of this gene is affected by food deprivation [51]; however, our study only found a significant difference in age, where *SCD* mRNA levels were much lower at 4 and 6 WOA than those at 2 and 8 WOA.

The amount of fat deposited in the body is regulated by a large array of factors, including energy composition in food, satiety of animals, and ability of cells to take up energy disposal due to movement. In farm animals, genes that determine population variations in fat deposition are largely unknown. An understanding of how adipose tissue expresses genes would help uncover the determining genes. The genes studied here are all expressed highly in chicken adipose tissue, indicating that they play a role in this tissue through various mechanisms.

## Figures and Tables

**Table 1:** Primers used for RT-qPCR analysis of gene expression.

Accession number	Gene	Forward sequence (5′)	Reverse sequence (3′)	Amplicon size (bp)
NW_003763826	IGFBP2	TGTGACAAGCATGGCTTGTACA	TCCACGCTGCCCATTCA	62
NM_204978.1	GREM1	CCATGAAGAAGGCTGCAACA	TGCATTGGCCATAGCAGAAC	58
NM_205509.2	FGFR3	GCTGATTT TGGCCTTGCTAGA	GGCAGCCGACCATTGGT	70
NW_003763661	ACBD7	AGATGTGAAAGGCAAAGCCAAA	TCATGGCATCCTCCTTCGAT	70
NM_205380	COL3A1	TTGTTCATTCTTGCCGTGTTTC	TCCTCCTAGGGCGTCCTGTT	62
M20816	TNC	CTGAGCAGATCTATGAGGAGCAAA	GGATAAGGATAAAGAAGACCAGTTGTG	70
NM_206951	EPHB2	CATGCCATGCACCACCAT	TTTCATTCACGCTGGAGATCAC	58
NW_003763812	SCD	GCGCTGCTCACATGTTTGG	TCTCCCGTGGGTTGATGTTC	56

**Table 2:** Mean bodyweight gains (BWG) of broiler chickens fed RSD and HCD.

	Age (week)								Total^1^
	1	2	3	4	5	6	7	8	
	Bodyweight gain (g)							
Treatment									
RSD male	44.23	147.72^b^	223.41^b^	402.64^b^	428.21^b^	536.96	649.52	537.78	**2970.53**
HCD male	42.23	160.43^a^	302.87^a^	445.72^a^	553.44^a^	537.09	601.78	524.93	**3148.49**
PSEM2	1.95	4.27	8.19	11.45	19.85	27.72	52.55	43.41	
RSD female	45.23	145.06^b^	227.40^b^	374.78	417.55	481.39^b^	516.07	513.79	**2721.27**
HCD female	46.66	160.11^a^	299.54^a^	397.34	459.32	564.60^a^	495.36	437.14	**2860.07**
PSEM2	2.47	4.41	8.24	13.06	21.81	27.42	46.01	48.78	

^a,b^Means within columns, within sex with no common superscript differ significantly (*P* < 0.05).

^1^Mean total eight-week bodyweight gains.

^2^Pooled SE of mean.

Dietary treatments: RSD (corn-soy; 3054 kcal/kg diet, ME); HCD (corn-soy; 3343 kcal/kg diet, ME).

**Table 3:** Bodyweight (g) of birds fed RSD and HCD.

Age (week)	Male				Female			
	RSD		HCD		RSD		HCD		
	*n*	Mean ± SE	*n*	Mean ± SE	*n*	Mean ± SE	n	Mean ± SE	
0	54	28.2 ± 0.25	59	28.4 ± 0.3	56	27.7 ± 0.4	57	27.8 ± 0.4	
1	44	70.6 ± 1.7	58	71.6 ± 1.7	50	69.3 ± 1.7^b^	37	76.2 ± 2.3^a^	
2	42	217.7 ± 6.9	54	228.1 ± 5	50	214.7 ± 4.9^b^	33	236.7 ± 8^a^	
3	37	434.1 ± 12.5^B^	48	522.6 ± 11.4^A^	44	438.6 ± 9.9^B^	28	542.8 ± 16.2^A^	
4	37	829.9 ± 23.7^B^	48	967.2 ± 18.4^A^	42	823.1 ± 14.6^B^	28	938.5 ± 29.8^A^	
5	32	1233.1 ± 39.5^B^	40	1508.4 ± 31.8^Ax^	36	1237.1 ± 27^b^	22	1373.5 ± 61.8^ay^	
6	31	1756.9 ± 58.8^B^	39	2049.4 ± 50.7^A^	35	1719.8 ± 29.5^B^	20	2052.3 ± 60^A^	
7	26	2284.8 ± 108.1^b^	31	2642.4 ± 100.2^a^	29	2218.3 ± 51.2^B^	13	2584.3 ± 82.4^A^	
8	25	2736.9 ± 167.6	31	3039.6 ± 139.7	23	2631 ± 99.2^b^	13	3041.8 ± 104.7^a^	

Note: *n*: number of birds in group. Superscripts a, b denote significant differences at *P* < 0.05 between diets at the same age within the same sex. Superscripts A, B denote significant differences at *P* < 0.01 between dietary groups at the same age within the same sex. Superscripts x, y denote significant differences at *P* < 0.05 between different sexes of the same diet and age.
